# The effect of Baduanjin exercise on health-related physical fitness of college students: A randomized controlled trial

**DOI:** 10.3389/fpubh.2022.965544

**Published:** 2022-12-01

**Authors:** Yu Ye, Fang Zhao, Shanshan Sun, Jian Xiong, Guohua Zheng

**Affiliations:** ^1^College of Nursing and Health Management, Shanghai University of Medicine and Health Sciences, Shanghai, China; ^2^Medical School of Chinese PLA, Chinese PLA General Hospital, Beijing, China; ^3^Department of Rehabilitation Medicine, The Second Medical Center, Chinese PLA General Hospital, National Clinical Research Center for Geriatric Diseases, Beijing, China

**Keywords:** college students, Baduanjin, exercise, physical fitness, randomized controlled trial

## Abstract

**Background:**

The continuous decline in the physical fitness of college students has become a serious social problem worldwide. Therefore, it is necessary to explore the effective method improving college students' physique. Previous studies have shown that Baduanjin exercise is beneficial in improving sleeping quality, mental health, body flexibility, and body physique. However, the evidence is unclear whether Baduanjin exercise can be recommended as an effective exercise to promote health-related physical fitness of college students.

**Methods:**

A total of 130 college students were recruited and randomly allocated to either the Baduanjin training or the control group at a ratio of 1:1. The students in the Baduanjin training group received a 12-week supervised Baduanjin exercise training intervention with a frequency of 1 h each day and 4 days per week, while those in the control group did not receive any specific exercise intervention and were informed to maintain their original lifestyle for 12 weeks. The outcomes of health-related physical fitness involving measurements of body flexibility, muscular strength, cardiopulmonary fitness, and body endurance were measured at baseline and after the 12-week intervention period. Mixed linear model was used to analyze the effect of the Baduanjin exercise intervention.

**Results:**

Mixed linear model analyses showed that the Baduanjin training group had a significant increase in the composite scores of health-related physical fitness compared to the control group from baseline to 12-week post-intervention with a medium effect size (*d* = 0.68, *P* = 0.006). Moreover, there were significant improvements in vital capacity, physical flexibility, 800/1,000 m endurance running, and body composition (measured by body mass index (BMI), fat mass and body fat ratio) at end of 12-week intervention in the Baduanjin training group. No adverse events were observed in this trial.

**Conclusion:**

Regular Baduanjin training may be an effective, safe exercise form to promote the health-related physical fitness of young adults.

**Trial registration:**

Chinese Clinical Trial Registry, ChiCTR-IOR-17013011. http://www.chictr.ogr.cn.

## Introduction

College students are in the golden age of acquiring knowledge and developing healthy behaviors, with strong plasticity (1). Most health-related behaviors are formed in late adolescence and youth (2). However, with the integration of digital technology within daily life, the sedentary lifestyle becomes prevalent among adolescents or college students globally (3, 4). As a result, their body shape, physical function, and quality of life are showing a downward trend (5). College students generally had insufficient rest time and long sitting time, leading to low motor function, manifested by an asymmetry of the body and poor trunk stability (6). Previous studies showed the average college students gained an estimated 1.55 kg during 4 years of study, especially during the first year of college (7), the prevalence of overweight and obesity in young college students in recent years has been increased in most counties (8). One study reported the prevalence of physical activity time <1 hour per day in college student of China was up to 82.5% for male students and up to 89.8% for females, respectively (9). It is well known that physical inactivity or low physical activity could lead to decreased physical fitness which had a positive association with multiple chronic diseases (10, 11). According to reported from National Student Physical Fitness Research, the health-related physical fitness of students in China had taken on a trend of gradual decline (12). While regular exercise is well-known to have beneficial effects on physical fitness for the general population.

Baduanjin is a widely used traditional Chinese Qigong exercise of combining body and mind. It is characterized by gentle and coherent postures, abdominal breathing in harmony with movement, and a mental state of meditation (13). Baduanjin only consists of eight movements and postures, but each of which involves specific parts and organs of the body, thereby enhancing its functions and bringing about health improvement (14). Increasing evidence have shown that Baduanjin training has a positive benefit in improving physical function, mental health, cognitive ability and reducing the risk of chronic diseases for older individuals with different health condition (15–17), but few studies focus on the effect of Baduanjin training for young adults. Our previous research found that regular practice of Baduanjin can improve the proprioception, flexibility and explosiveness of the lower limbs of college students (18). Therefore, we hypothesized that regular Baduanjin training would improve the physical physique of young college students. In this study, we conducted a randomized controlled trial to examine the effect of regular Baduanjin training on the health-related physical fitness of college students.

## Methods

### Study design

This was a randomized controlled trial to investigate the effect of 12-week Baduanjin training for health-related physical fitness of college students. A total of 130 eligible college students from Shanghai University of Medicine and Health Sciences (SUMHS) were recruited and randomly allocated to either Baduanjin exercise training group or control group with a ratio of 1:1. The indicators of health-related physical fitness were measured at baseline and end of 12-week intervention. This trial was carried out in accordance with the declaration of Helsinki and approved by the Ethics Board of SUMHS (approval number: 2017ZGH). All participants provide written informed consent prior to participation. The design of the study was detailed in the published protocol (19).

### Participant

Participants were recruited from SUMHS through the campus radio, flyers and WeChat between May 1 and October 1, 2018. Participants were eligible if they met the following criteria: full-time college students aged 16–25 years; freshmen or sophomores; and provided the informed consent. Those, engaging in a long-term regular practice of Baduanjin or other exercise type; being in a member of martial arts, dance, aerobics, Sanda, Taekwondo and other associations; or suffering from severe cardiovascular disease, musculoskeletal system disease or other exercise contraindications, were excluded.

### Randomization and masking

The random allocation sequence was produced by an independent statistician using the PLAN program of the SAS software version 9.1 (SAS Inst., Cary, NC, USA), and managed in a research assistant who did not participate in the recruitment. After the baseline assessments, the research assistant informed each eligible participant his or her group (Baduanjin training or control group) through the WeChat. Although it was difficult to blind the participants and exercise coaches, we masked the group information to outcome assessors and statistician.

### Interventions

#### Baduanjin training group

**Figure 1:**
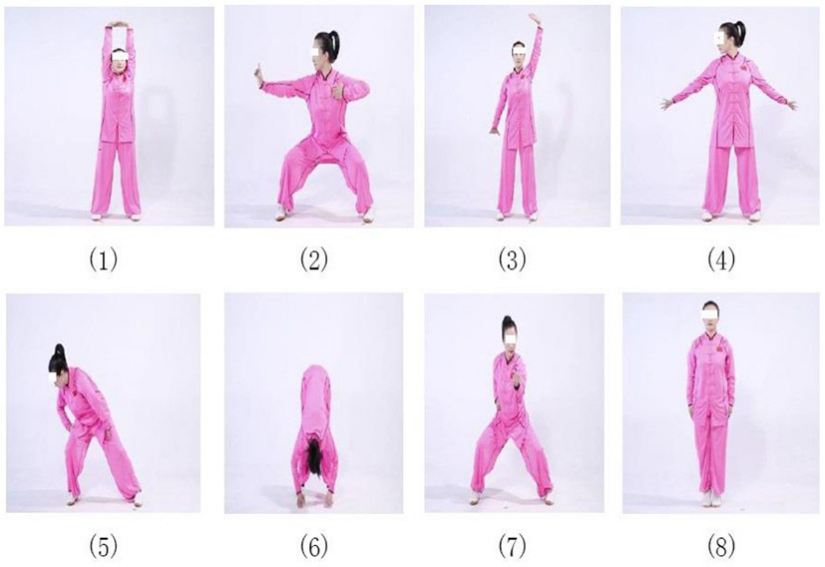
The Baduanjin exercise movements and postures.

Participants in the Baduanjin exercise training group received 12-weeks Baduanjin training at the gymnasium of the university with a frequency of 4 days 1 week and 1 h 1 day. The training scheme originated from Health Qigong- Baduanjin, published by the General Administration of Sport of China in 2003 (20). Participants who were allocated to the Baduanjin exercise group were gathered in the university stadium for Baduanjin practicing according to intervention scheme, in which 1 h Baduanjin exercise training consisted of 5 min warm-up, 5 min cooling-down, and 4 sets of Baduanjin practices with 1–2 min break between two sets. Baduanjin training was instructed by two qualified coaches who had engaged in the physical education over 5 years. The whole set of Baduanjin exercise consists of eight movements and postures (Figure 1). Two qualified coaches were employed to instruct the participants' correct Baduanjin postures and supervised their practice during the 12-week intervention period.

#### Control group

Participants in the control group did not receive any specific exercise training, and were informed to maintain their original lifestyle during the 12-week intervention period.

Additionally, all participants were asked to record their daily physical activities by a step counter in their mobile phones during the 12-week period.

### Outcome assessment

Primary and secondary outcomes were measured at baseline and the end of 12-week intervention period by physical education teachers who did not participate in the implementation of this trial.

Primary outcome was the health-related physical fitness, which consist of 7 indicators on physical function including body mass index (BMI), vital capacity (VC), 50 meters running, body anteflexion in sitting position (BAISP), standing long jump (SLJ), pull-ups (male) or sit-ups (female), and 1,000 m running (male) or 800 meters running (female) according to China's National Student Physical Health Standard (2014 Revision) (21). The health-related physical fitness was evaluated by using a composite scores which could be calculated as the summation of the points of each above indicators multiplied by their corresponding weight (i.e., a composite scores of health-related physical fitness = 15% BMI points + 15% VC points + 20% 50-m running points + 10 BAISP points +10 SLJ points +10% pull-up (male) or sit-up (female) points +20% 1,000 m (male) or 800 meters (female) running points). The scoring standard and weight of each indicator and their measurement methods were described in the published protocol (19).

The secondary outcomes included handgrip strength, cardiorespiratory endurance, body composition, and all of single indicators of health-related physical fitness. Their measurement tools and method were described in the published protocol (19).

### Sample size and statistical analysis

The sample size was estimated based on the composite scores of health-related physical fitness reported previous study data, where the mean and standard deviation of the composite scores in the health-related physical fitness of college students were measured as 55 points and 12.5 points, respectively (22). A total of 130 samples were necessary to have 80% power to detect a difference in the composite scores of health-related physical fitness of 5.0% between Baduanjin training and control group after intervention with a maximum loss to follow-up of 20% (23).

Baseline characteristics, primary and secondary outcomes at baseline and after intervention between groups were compared using the *t*-test or Mann-Whitney test for continuous variables and Pearson's χ2 test for categorical variables. Between-group effect size were calculated using Cohen's d, in which effect sizes of 0.2, 0.5, and 0.8 were considered small, medium, and large effects, respectively (24). The intervention effect between the Baduanjin training group and control group from baseline to 12-week post-intervention was analyzed by using the mixed linear models with restricted maximum likelihood. The missing data were imputed using a multiple imputation method. All analysis was performed using SPSS 21.0 (IBM, Chicago, IL, USA) software. Statistical significance is defined as a two-sided with a 5% level of statistical.

## Result

### Baseline characteristics of participants

**Figure 2:**
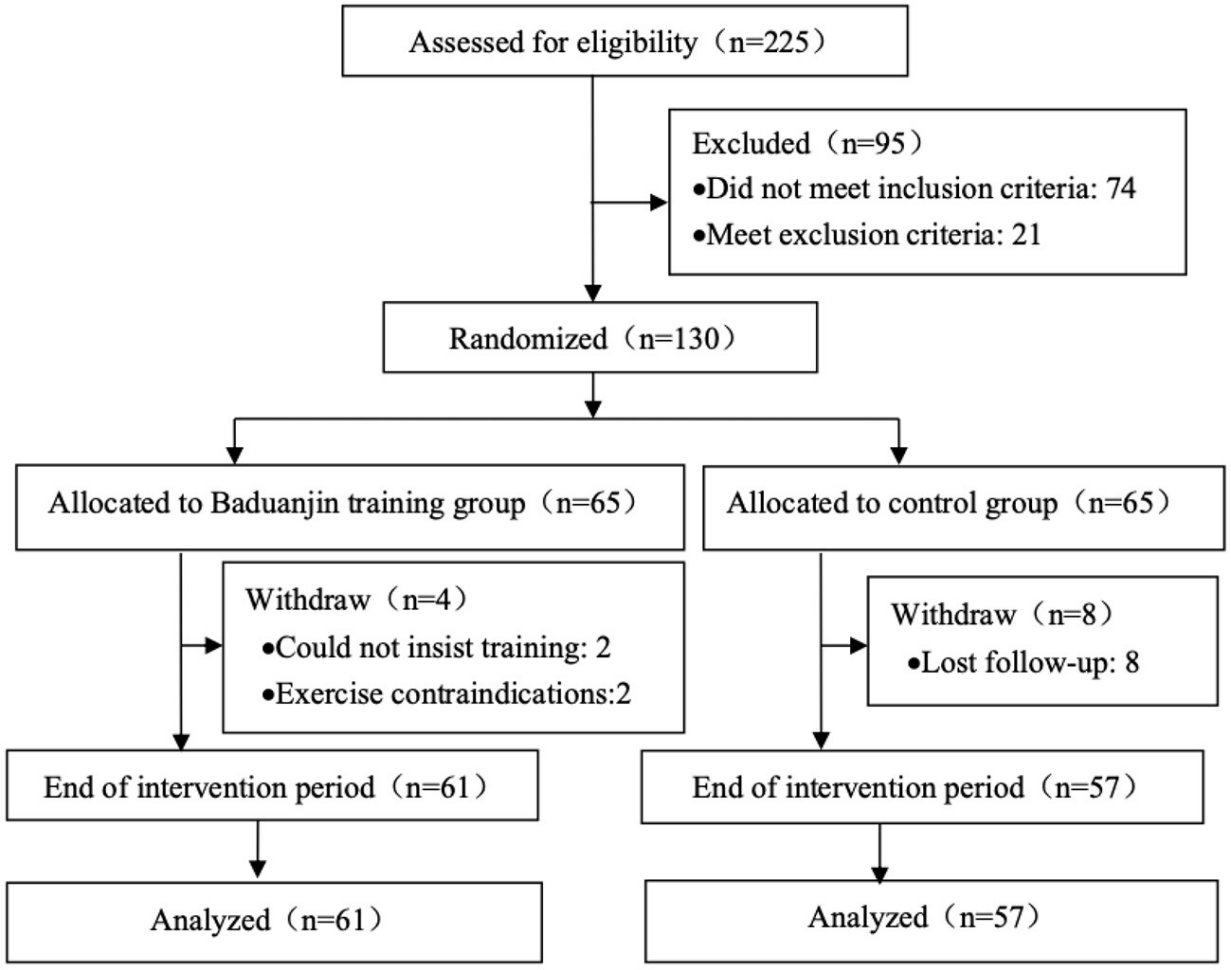
Participant flow diagram.

Figure 2 illustrates the participants' flow. 225 volunteers from the first or second grade undergraduates in SUMHS were assessed the eligibility. 130 students were eligible, and were randomly allocated into the Baduanjin training group (*n* = 65) or the control group (*n* = 65). During the intervention period, 12 participants (4 in the Baduanjin training group and 8 in the control group) dropped out this trial. Of 61 participants in the Baduanjin training group with the completed 12-week Baduanjin training, 35 participants had over 85% attendance rate (actual training days/ plan training days), and other participants possessed 75–85%. In 118 participants who were included in analysis, 93.2% of them were female students; the average age of participants was 18.2 years old. Baseline characteristics between groups including sex proportion, the average of age, BMI and baseline exercise time had no significant difference (Table 1).

**Table 1:** Baseline characteristics of the participants [mean (SD)].

**Characteristics**	**BDG**	**CG**	**t/χ^2^ value**	***P*-value**
	***n* = 61**	***n* = 57**		
Age (years)	18.90 (1.02)	18.80 (0.99)	0.539	0.890
Gender (Male/Female, *n*)	5/56	3/54	0.401	0.528
BMI (kg/m^2^)	21.55 (2.95)	20.97 (3.22)	1.021	0.154
Baseline exercise time^*^ (min)	35.30 (20.81)	32.25 (18.70)	0.835	0.269

* Baseline exercise time was the self-reported mean time of undergone moderate-vigorous exercise in the last 1 month.

BDG, Baduanjin training group; CG, control group; CI, confidential interval.

### Duration of activity during intervention

The average time of sedentary, low intensity activities and high intensity activities between groups were not statistically different but the average time of moderate intensity activities in the Baduanjin training group were higher than the control group during the 12-week intervention period (Table 2).

**Table 2:** The comparison of average activities time between two groups during the 12-week intervention period (hours).

**Characteristics**	**BDG**	**CG**	**t/χ^2^ value**	***P*-value**
	***n* = 61**	***n* = 57**		
Sedentary time (h)	8.4 (2.1)	8.7 (2.5)	0.707	0.481
Low intensity activities time (h)	1.85 (0.9)	1.75 (0.92)	0.597	0.551
Moderate intensity activities time (h)	1.16 (0.35)	0.65 (0.37)	7.695	<0.001
High intensity activities time (h)	0.25 (0.22)	0.23 (0.28)	0.433	0.665

BDG, Baduanjin training group; CG, control group; CI, confidential interval.

Sedentary time: defined as the time of sitting down with waking state, such as attending classes, reading, playing computer, etc; Low intensity activities: defined as those with energy consumption <3.0 Metabolic Equivalents (METs) including walking, general housework, mild stretching exercise, etc; Moderate intensity activities: defined as those with energy consumption range from 3.0 to 6.0 METs including Baduanjin training, brisk walking, jogging, cycling, stretching exercise, etc; High intensity activities: defined as those with energy consumption over 6.0 METs including running, playing ball games, mountaineering, etc.

### Primary outcome

Table 3 presents the statistical summary of the composite scores of health-related physical fitness from the baseline to 12-week post-intervention. The composite scores between two groups were similar (*P* > 0.05) at baseline, but this scores in Baduanjin training group were significantly higher than the control group (*P* < 0.05) with a medium effect size (*d* = 0.68) at 12-week post-intervention. The linear mixed model analysis found that the composite scores were increased in the Baduanjin training group, and decreased in the control group from the baseline to 12-week post-intervention assessment. Moreover, the significant group × time difference was found (*P* = 0.006).

**Table 3:** The comparison of the composite scores of health-related physical fitness between the two groups (percentile system).

**Outcome**	**BDG (*n* = 61)**	**CG (*n* = 57)**	**Comparisonbetween groups**	**Between-group effect size**	**Time × group interaction**
	**Mean ±SD**	**Mean ±SD**	***t/P*-value**	**Cohen's d 95% CI**	***F/P*-value**
Baseline	70.9 ± 8.3	68.2 ± 10.1	1.59/0.114		
12-week intervention	71.3 ± 9.3	64.5 ± 10.1	3.81/ <0.001	−0.68 (−1.05, −0.31)	7.70/0.006

Values are expressed in mean ± standard deviations; BDG, Baduanjin training group; CG, control group; CI, confidential interval.

### Secondary outcomes

Table 4 presents the statistical summary of secondary outcomes including the single indicator of health-related physical fitness, handgrip strength, cardiopulmonary fitness and body composition measures from the baseline to 12-week post-intervention. All measures but body anteflexion in sitting position were balanced at the baseline. After 12-week intervention period, the vital capacity, body anteflexion in sitting position, 1000 or 800 m running in the Baduanjin training group were significantly better than the control group, with low (*d* = 0.39), medium (*d* = 0.53) and high (*d* = 0.82) effect sizes. The mixed linear model analysis demonstrated that the BMI, 1,000/800 m running time, fat mass and body fat were reduced significantly more in the Baduanjin training group than the control group from the baseline to 12-week post-intervention, with the significant group by time effect (*P* = 0.006, 0.001, 0.012, and 0.036, respectively).

**Table 4:** The comparison of the single indicator constructing health-related physical fitness, cardiopulmonary fitness, handgrip strength and body composition between two groups.

**Outcome**	**BDG** **(*n* = 61)**	**CG** **(*n* = 57)**	**Comparison between groups** **t/*P*-value**	**Between-group effect size** **Cohen's d 95% CI**	**Time × group interaction** **F*/P*-value**
	**Mean ±SD**	**Mean ±SD**			
**The specific indicators constructing health-related physical fitness**					
**BMI**					
Baseline	21.6 ± 2.9	21.0 ± 3.2	1.06/0.288		
12-week intervention	21.0 ± 2.8	20.8 ± 3.4	0.35/0.727	−0.06 (−0.42, 0.30)	7.73/0.006
**Vital capacity (ml)**				
Baseline	2,648.2 ± 706.6	2,516.0 ± 581.8	1.11/0.271		
12-week intervention	2,544.6 ± 707.9	2,277.9 ± 662.4	2.11/0.037	−0.39 (−0.76, −0.02)	1.724/0.192
**50 meters running (second)**		
Baseline	8.8 ± 1.1	9.0 ± 0.8	1.12/0.264		
12-week intervention	9.3 ± 0.9	9.3 ± 0.9	0.18/0.857	−0.01(−0.36, 0.36)	1.23/0.270
**Body anteflexion in sitting position (cm)**	
Baseline	17.4 ± 8.9	13.5 ± 6.4	2.72/ <0.01		
12-week intervention	18.3 ± 7.4	14.5 ± 7.0	2.86/ <0.01	−0.53 (−0.89, −0.16)	0.023/0.880
**Standing long jump (cm)**		
Baseline	169.1 ± 27.4	161.1 ± 26.5	1.61/0.110		
12-week intervention	168.2 ± 34.3	166.5 ± 35.3	0.27/0.791	−0.05 (−0.41, 0.31)	1.78/0.185
**Pull-ups/ sit-ups (numbers/min)**		
Baseline	28.4 ± 13.0	27.5 ± 11.2	0.40/0.688		
12-week intervention	31.9 ± 10.7	28.8 ± 9.8	1.64/0.104	−0.37(−0.74, −0.01)	0.332/0.631
**1000/800 meters running (min)**		
Baseline	4.0 ± 1.1	3.8 ± 0.9	1.08/0.284		
12-week intervention	3.8 ± 0.6	4.3 ± 0.6	4.52/ <0.01	0.82 (0.44, 1.19)	15.42/ <0.001
**Cardiopulmonary fitness (**Step test index)			
Baseline	54.7 ± 8.2	57.9 ± 8.9	2.03/0.04		
12-week intervention	56.4 ± 10.6	55.6 ± 9.8	0.42/0.672	−0.07 (−0.43, 0.29)	3.344/0.07
**Handgrip strength (kg)**				
Baseline	27.1 ± 9.1	26.8 ± 8.3	0.51/0.614		
12-week intervention	26.8 ± 10.8	27.9 ± 8.0	0.63/0.533	0.11 (−0.25, 0.47)	0.453/0.502
**Body composition**					
**Fat mass (kg)**					
Baseline	15.8 ± 5.5	15.8 ± 6.7	0.01/1.00		
12-week intervention	14.8 ± 5.3	15.6 ± 6.4	0.74/0.459	0.14 (−0.22, 0.50)	6.569/0.012
**Body fat (percentage, %)**				
Baseline	27.6 ± 6.4	27.3 ± 7.6	0.23/0.816		
12-week intervention	26.3 ± 6.5	27.1 ± 6.8	0.65/0.515	0.12 (−0.24, 0.48)	4.522/0.036
**Fat free mass (kg)**					
Baseline	40.6 ± 5.7	41.0 ± 5.0	0.40/0.686		
12-week intervention	40.6 ± 5.75	40.8 ± 5.1	0.19/0.842	0.03 (−0.33, 0.39)	0.176/0.675
**Lean body mass (kg)**					
Baseline	38.1 ± 5.4	38.5 ± 4.7	0.43/0.669		
12-week intervention	38.1 ± 5.4	38.3 ± 4.8	0.21/0.832	0.04 (−0.32, 0.40)	0.148/0.701

Values are expressed in mean ± standard deviations; BDG, Baduanjin training group; CG, control group; CI, confidential interval.

### Adverse events

During the intervention period, no adverse events related to the intervention were found. Two participants in the Baduanjin training group withdrawn out the trial because their legs were sprained when they played basketball game.

## Discussion

To the best of our knowledge, this was the first RCT to explore the effects of the traditional Chinese mind-body exercise-Baduanjin on the health-related physical fitness for the young college students. The results showed that compared with the control group without special exercise intervention, 12-week regular Baduanjin training could significantly increase the composite scores of the health-related physical fitness assessment of college students. The effect sizes at the end of the intervention were medium, thereby indicating a notable clinical effect. For the special measures on the health-related physical fitness, 12-week Baduanjin training could significantly reduce the BMI, fat mass and body fat ratio, shorten the 1,000/800 m running time, and increase vital capacity and body anteflexion in sitting position compared with no special exercise intervention control, and with the medium and high effect size for body anteflexion in sitting position and 100/800 meters running. Moreover, no adverse events related-Baduanjin exercise were observed during 12-week intervention period. It suggests that the Baduanjin exercise is safe for the college students.

As one of traditional Chinese mind-body exercises, Baduanjin with the characteristics of relaxation techniques, meditation, and breathing regulation, can unite the body and mind to positively influence physical function, psychological status, and symptoms (25). When practicing Baduanjin exercise, practicers need to keep their body a steady gravity center. Then take the lumbar spine as the axis to drive their four limbs movement, while alternately change the muscle tension and relaxation at different parts of the body. At same time, the mind, body, and breath are required to be smooth and unstrained (26). Numerous studies have already proven that Baduanjin is a popular community exercise to promote health in China, and especially suitable for older adults (13, 17, 27). The benefit of regular Baduanjin training could be expressed through adjusting breathingyy to make the process of smoother, unifying mind and breathing, strengthening muscles and tendons to make the body more flexible and the unison of mind and body (25). Current study found that compared to no special exercise intervention control, 12-week Baduanjin training could obviously increase the composite scores of health-related physical fitness of young college students, and with a medium effect size. Health-related physical fitness refers to the ability of the cardiovascular, lungs and muscles to perform optimally (28). It is a diversified structure that covers the characteristics of each body composition, namely body mass index, muscle strength, cardiopulmonary function, balance, coordination ability, speed and agility (29). These ingredients are closely related to the maintenance of individual's healthy physiological function (30–32). One previous study conducted in community healthy adults aged from 20 to 59 years reported that 16-week Baduanjin training could significantly improve their physical flexibility and subcutaneous adipose accumulation, therefore be helpful to promote the physical fitness and health of adults (33). Another RCT among the homebound elderly also found 12-week (3 months) Baduanjin training could significantly increase forced vital capacity, maximum voluntary ventilation, activities of daily living, and self-reported health status (34). Our findings are consistent with their previous research results, and indicate that regular Baduanjin training is helpful for improving the health-related physical fitness of college students.

For the specific indicators for constructing health-related physical fitness, the results of this trial shown that 12-week Baduanjin training could significantly reduce BMI and 1,000/800 m running time, and increase vital capacity and body anteflexion in sitting position of college students. These indicators express the ability of body constitution, pulmonary function, endurance and flexibility of health-related physical fitness. Several studies have reported that Baduanjin training could reduce the BMI for the different population (33, 35). One previous systematic review from 19 RCTs also provided useful evidence that Baduanjin practice could improve physical flexibility, but it was necessary to confirm the effect on endurance and pulmonary function (36). The findings of this study provided a strong support for this evidence. Our previous RCT found that 12-week Baduanjin training had an advantage for college students on improvement of cardiorespiratory endurance and flexibility (18). In addition, current study also found that 12-week Baduanjin training could decrease the fat mass and body fat ratio, therefore it might have a beneficial effect for body composition. This results also support the effect of Baduanjin for BMI. The step test is the most intuitive and simple exercise test for assessing cardiorespiratory fitness (37), our previous RCT found that 12-week Baduanjin training could significantly increase the step test index of college students (18), while current RCT did not draw the similar findings, but we found the step test index in the Baduanjin training group were increased by an average of 1.7 from baseline to 12-week post-intervention, while it was decreased by 2.3 in the control group, and the comparison between groups was nearly statistically significant (*P* = 0.07). Otherwise, this study did not find the significant difference between groups in handgrip strength, other indicators constructing health-related physical fitness include standing long jump, 50 m running time and pull-ups/ sit-ups. Therefore, more RCTs in future are necessary to confirm the effect of Baduanjin training on health-related physical fitness of college students.

### Strengths and limitations

Study strengths include the rigorous randomized, parallel-controlled design, a relatively large samples, the blinded outcome assessment and statistical analysis, the better adherence to intervention, and high quality of research control that the qualified Baduanjin instructors were invited to guide participants' training. Usually, Baduanjin is recommended to the community dwelling older population with or without chronic diseases as a safe and effective way to promote health (13, 27). This trial focus on effect of regular Baduanjin training for young college population. Therefore, this study may provide a reference for the application of Baduanjin exercise in young people.

Several limitations should be considered in this trial. First of all, due to the samples from the same university, the relatively homogenous participants would limit generalizability. Second, because it is not feasible to blind participants and exercise instructors in the trial with the exercise intervention, therefore the subjective bias from participants of Baduanjin training group may be unavoidable. Third, although some references addressed Baduanjin as a moderate intensity exercise (25, 38), no measures were used to evaluate its real exercise intensity in current trial, which may result in some unpredictable confounding influence. Fourth, this trial only compared the effect of Baduanjin exercise and no exercise on physical fitness of college students, lack of the design to compare the effect of Baduanjin exercise and other conventional exercise type on the physical fitness in college students. Thus, this may be a design fault, and should be considered in future study. Finally, the intervention period was limited to only 12 weeks Baduanjin training, which may not be enough to demonstrate all the protective effects of Baduanjin exercise.

## Conclusions

This study demonstrates that 12-week regular Baduanjin training may significantly improve health-related physical fitness in young college population, involving in increasing physical flexibility and endurance, as well as reducing BMI and body fat mass. Our finding indicates that regular Baduanjin exercise may be an effective, safe exercise form to promote the health-related physical fitness of young adults.

## Data availability statement

The datasets presented in this study can be found in online repositories. The names of the repository/repositories and accession number(s) can be found below: & Chinese Clinical Trial Registry, ChiCTR-IOR-17013011. http://www.chictr.ogr.cn.

## Ethics statement

The studies involving human participants were reviewed and approved by Ethics Board of Shanghai University of Medicine and Health Sciences (approval number 2017ZGH). Written informed consent to participate in this study was provided by the participants' legal guardian/next of kin.

## Author contributions

Conceived and designed the trial: GZ. Performed the trial: FZ, SS, and JX. Analyzed the data and write the manuscript: YY and GZ. All authors approved final version.

## Funding

This research is funded by the Key Project of School Sport Scientific Research in Shanghai (HJTY-2017-A07).

## Conflict of interest

The authors declare that the research was conducted in the absence of any commercial or financial relationships that could be construed as a potential conflict of interest.

## Publisher's note

All claims expressed in this article are solely those of the authors and do not necessarily represent those of their affiliated organizations, or those of the publisher, the editors and the reviewers. Any product that may be evaluated in this article, or claim that may be made by its manufacturer, is not guaranteed or endorsed by the publisher.
